# Atypical Neuroleptic Malignant Syndrome in the Setting of Quetiapine Overdose: A Case Report and Review of the Literature

**DOI:** 10.7759/cureus.12602

**Published:** 2021-01-10

**Authors:** Sheila D Hernandez, Dario A Marotta, Ravitej Goteti

**Affiliations:** 1 Department of Internal Medicine, Southeast Health, Dothan, USA; 2 Department of Research, Alabama College of Osteopathic Medicine, Dothan, USA; 3 Department of Neurology, Division of Neuropsychology, University of Alabama, Birmingham, USA

**Keywords:** neuroleptic malignant syndrome, overdose, atypical antipsychotic

## Abstract

Neuroleptic malignant syndrome (NMS) is a rare and life-threatening emergency. The condition is largely iatrogenic and is often precipitated by medications such as antipsychotics. First-generation antipsychotics are more likely to cause NMS than second-generation antipsychotics. The literature lacks an objective measure for NMS diagnosis. Instead, the diagnosis relies largely on the recognition of characteristic symptoms in the presence of an inciting medication. Additional challenges exist with concomitant disease processes and toxicities that may distort the clinical picture. Here, we report a case of a 44-year-old Caucasian man who presented with atypical NMS in the setting of quetiapine overdose. The patient remained uncharacteristically afebrile throughout his admission. Although the patient recovered, extended delays in identification and management can contribute to an increased risk of morbidity and mortality.

## Introduction

Neuroleptic malignant syndrome (NMS) is a rare and potentially lethal constellation of symptoms [1-2]. Neuroleptic medications, particularly first-generation antipsychotics and certain antiemetics, have been shown to induce symptoms [3-5]. However, induction by second-generation antipsychotics, such as quetiapine, is less likely. Definitive diagnostic criteria do not exist for NMS, although hyperthermia, altered mental status, muscle rigidity, elevated creatine kinase (CK), and autonomic instability in the setting of newly introduced or increased doses of neuroleptic medications should prompt a high index of suspicion [3]. Unfortunately, not all cases of NMS present with hallmark signs and symptoms. NMS in the setting of alcohol or drug overdose can obfuscate the clinical picture. The risk of heightened morbidity and mortality requires prompt identification and persistent monitoring [3,5]. Here, we present a 44-year-old male with an atypical presentation of NMS in the setting of quetiapine overdose.

## Case presentation

A 44-year-old Caucasian male with a past medical history pertinent for attention-deficit hyperactivity disorder and major depressive disorder with psychotic symptoms controlled with quetiapine (30 mg daily), fluoxetine (40 mg daily), and dextroamphetamine salts (30 mg daily) presented to the emergency department via ambulance after being found on the floor of his home. The patient has a known history of suicide attempts. Upon examination, the patient was obtunded, disheveled, speaking unintelligibly, and only responsive to tactile stimuli. The patient’s vital signs included a blood pressure of 119/83, pulse of 108, temperature of 96.6 F, respirations of 15, and oxygen saturation of 96% on room air. The patient was found in the presence of an empty bottle of quetiapine. His mother was unsure as to how long he had been on the floor. However, she advised that he might have consumed excessive amounts of alcohol the night before.

Further workup revealed a urine toxicology screen positive for amphetamines and an elevated blood alcohol concentration (70 mg/dL; reference range 0-10 mg/dL). Acetaminophen and salicylate levels were within normal limits. Serology was remarkable for leukocytosis (15.8x10-3/uL; reference range 4.5-10.0x10-3/uL), a liver profile with slight elevation of aspartate transaminase (50 IU/uL; reference range 13-39 IU/L), and elevated lactate (8.04 mmol/L; reference range 0.90-1.70 mmol/L). Urinalysis was positive for a few bacteria and 2+ blood. A basic metabolic panel was impressive for hyperkalemia (l6.4 mEq/L; reference range 3.5-5.1 mEq/L) and elevated creatinine (2.6 mg/dL; reference range 0.60-1.30 mg/dL) in an otherwise healthy kidney. Phosphorus and phosphokinase were also elevated. Arterial blood gas revealed a high anion gap metabolic acidosis. A summary of the patient’s acute workup, with notable findings, can be found in Table 1.

**Table 1:** Summary of Pertinent Laboratory Values at Time of Admission ABG = Arterial blood gas; CBC = Complete blood count; CMP = Comprehensive metabolic panel; UA = Urinalysis; S = Serology; T = Toxicology; H = Patient value above the upper limit of normal; N = Patient value within reference range; L = Patient value below the lower limit of normal

Laboratory type	Analyte	Patient value	Reference value
T	Amphetamines	Positive	Negative
T	Blood alcohol concentration	70.0 H	0.0-10.0 mg/dL
CBC	Leukocytes	15.8 x 10^-3^ H	4.5-10.0x10^-3^/uL
CMP	Aspartate transaminase (AST)	50 H	13-39 IU/L
CMP	Alanine aminotransferase (ALT)	22 N	7-52 IU/L
CMP	Lactate	8.04 H	0.90-1.70 mmol/L
CMP	Phosphorus	6.4 H	2.5-5.0 mg/dL
CMP	Creatinine	2.1 H	0.60-1.30 mg/dL
ABG	pH	7.452 H	7.350-7.450
ABG	pCO2	25.0 L	35.0-45.0 mm Hg

**Figure 1:**
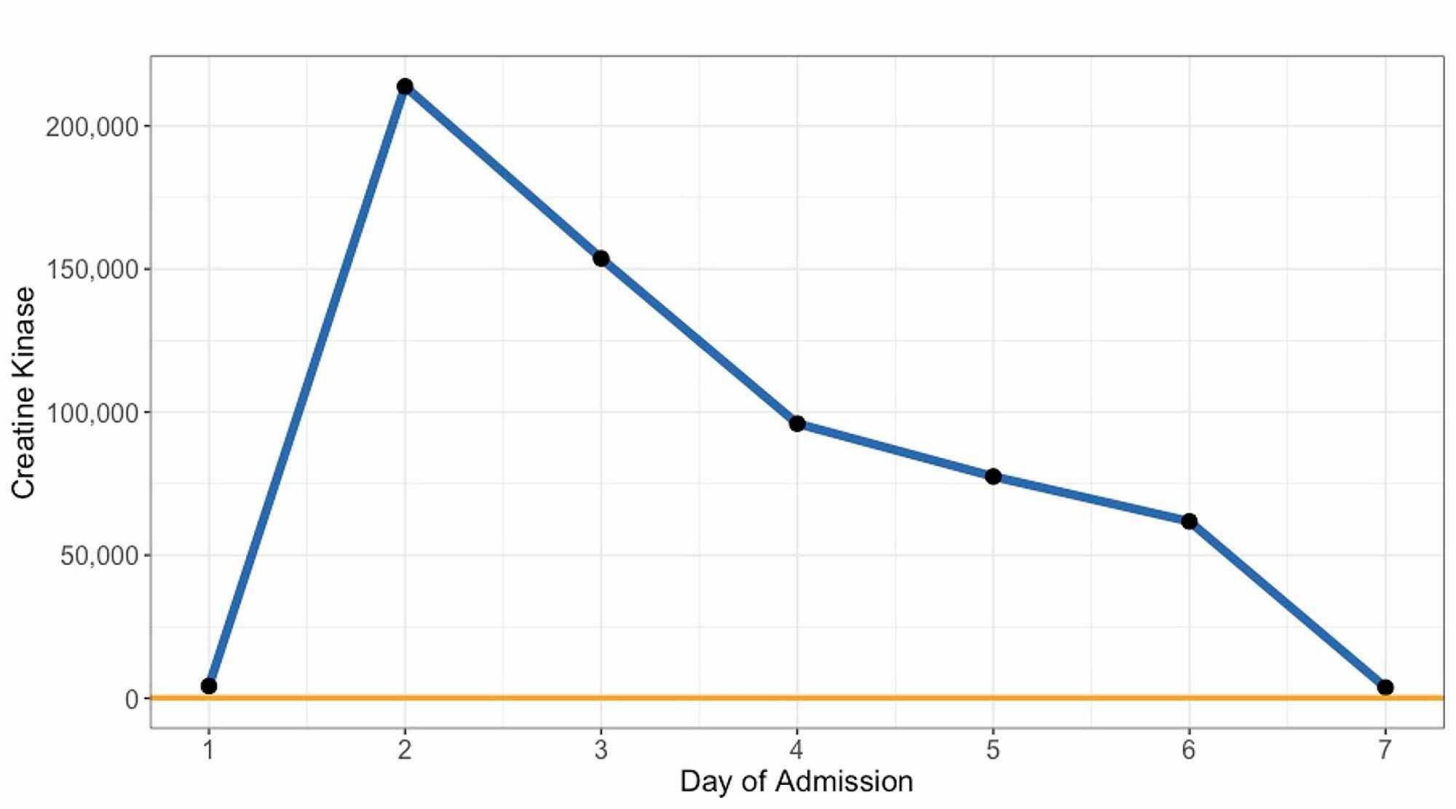
Longitudinal Creatine Kinase Levels During Admission Blue = Patient creatine kinase levels; Orange = Reference values

The patient was admitted to the internal medicine service with the working diagnosis of acute metabolic encephalopathy secondary to alcohol intoxication or amphetamine overdose with concomitant rhabdomyolysis. The patient was carefully monitored on an inpatient basis. By day two of admission, the patient's creatine kinase (CK) dramatically increased from 4,281 IU/L to 213,800 IU/L (reference range 30-223 IU/L). A longitudinal scatter plot of the patient’s CK over the course of admission is provided in Figure 1. Creatinine also increased despite fluid hydration. His mentation failed to improve, and he became aggressive, requiring physical restraints. The patient remained afebrile throughout his admission. He was persistently tachycardic with stable oxygen saturation (95%-100% on room air). Although the patient presented afebrile, all other signs and symptoms suggested NMS. The patient was transferred to the intensive care unit. Psychotropic medications were withheld, with the exception of lorazepam as needed for agitation. The patient’s normal basal temperature, mild muscle rigidity, and elevated liver enzymes precluded the use of dantrolene at that time.

**Figure 2:**
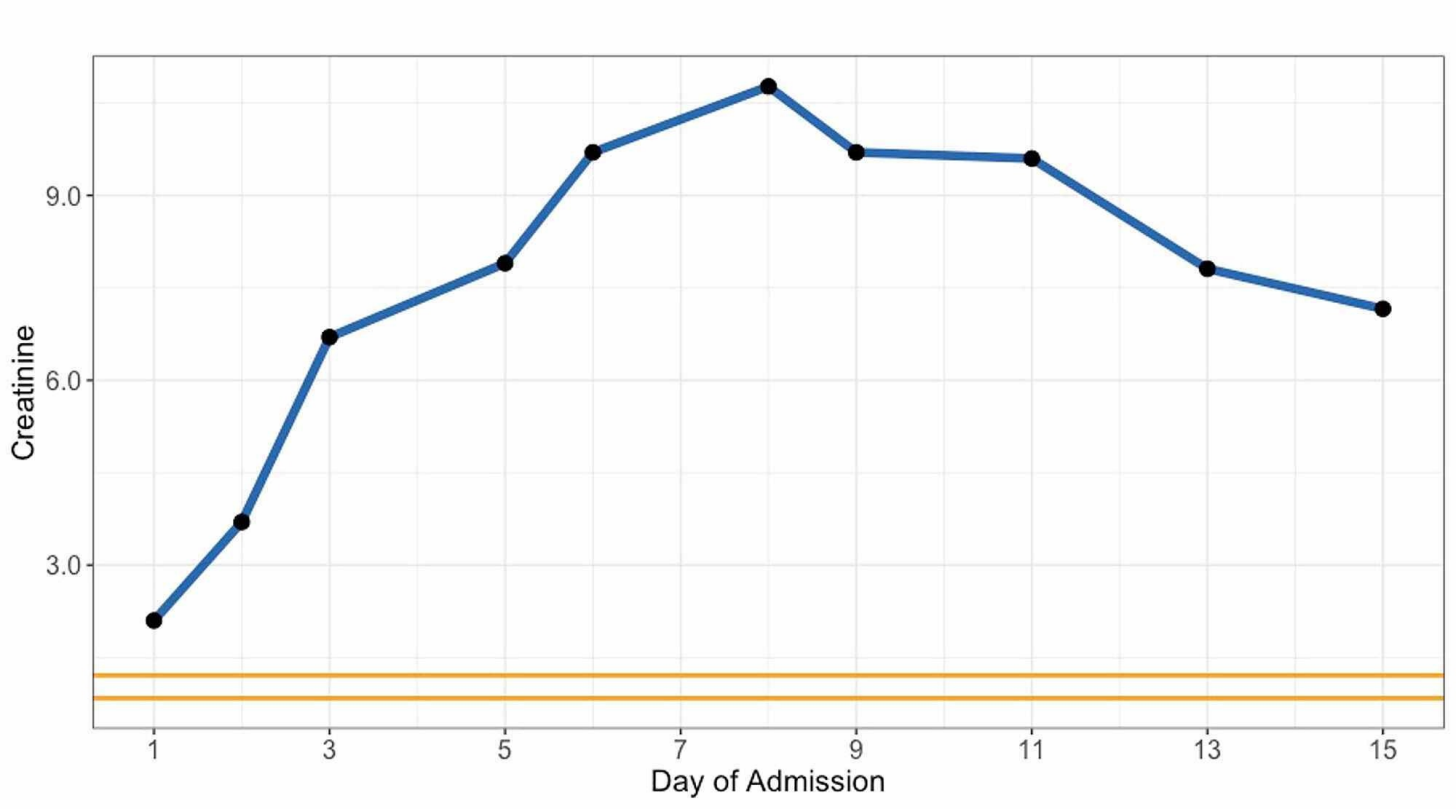
Longitudinal Creatinine Levels During Admission Blue = Patient creatinine levels; Orange = Reference values

Despite aggressive fluid resuscitation, the patient experienced acute renal failure with a creatinine increasing steadily from 2.10 mg/dL on admission to 7.2 mg/dL on day two (Figure 2). Other metabolic derangements were evident, including worsening metabolic acidosis and hyperkalemia. The patient underwent hemodialysis on post-admission day two and his clinical course improved without incident over the next 18 days. The patient was discharged requiring routine dialysis for the foreseeable future.

## Discussion

Neuroleptic malignant syndrome (NMS) is a rare and life-threatening emergency leading to mortality in 30%-50% of unmanaged patients [3-4]. Prompt identification and continuous monitoring are necessary to reduce the risk of morbidity and mortality. Recognition can be challenging and complex, requiring careful consideration of symptoms that may overlap with a myriad of concomitant disease states. In this case, we report a middle-aged man presenting to the emergency department in an obtunded state. The patient had a prior history of suicide attempts and his past medical history was significant for depression with psychotic features controlled with more than one psychotropic medication. Although the totality of the circumstances suggested overdose toxicity, the patient’s symptoms were not fully characteristic of an overdose with any one of his prescription medications alone.

Reports of quetiapine-induced NMS are limited, however, a case report analysis of 19 cases was conducted by Murri and colleagues, which revealed pooled clinical features with respect to quetiapine-induced NMS to include: 1) tachycardia, tachypnea, diaphoresis, and tremor in 100% of patients; 2) hyperpyrexia and rigidity in 92.3% of patients; 3) dysautonomia in 90% of patients; 4) mental status change in 85.7% of patients; and 5) fever in 78.9% patients [6]. Commensurate with this study, a set of diagnostic criteria proposed by Levenson in 1985 suggested similar features were characteristic of NMS and categorized them into major criteria (fever, rigidity, elevated creatine phosphokinase (CPK)) and minor criteria (tachycardia, abnormal arterial pressure, altered consciousness, diaphoresis, and leukocytosis) [2]. The presence of three major or two major and four minor signs suggests a high probability of NMS. In this case, the patient displayed moderate rigidity, elevated CPK, tachycardia, altered consciousness, leukocytosis, and abnormal arterial pressure, thereby satisfying two major and four minor criteria. However, these symptoms overlap with other more common conditions, such as malignant hyperthermia, in which full rigidity and mental status change are characteristic. Serotonin syndrome is also accompanied by similar symptoms, such as high fever, tachycardia, and confusion, while other well-reported entities, such as central nervous system (CNS) infections and toxins, can also be present in a similar manner and may complicate diagnosis and treatment [1,5].

Symptom onset and inciting medications can help identify or rule out NMS. For instance, NMS presents with an idiosyncratic onset, commonly developing between 24 hours and one month of newly introduced neuroleptic medication. Sixty-five percent of patients experience symptoms within one week, with a modicum of cases documenting symptoms within 24 hours [3]. First-generation antipsychotics (i.e., haloperidol, chlorpromazine, fluphenazine) are more commonly associated with NMS than second-generation antipsychotics [7]. Although, almost any medication that produces fulminant reductions in dopaminergic activity (either by blocking D2 receptors or abrupt withdrawal of D2 receptor stimulation) may cause suggestive symptoms [4-5]. Symptoms can arise from the initial introduction of antipsychotic therapy but have also been reported in patients with longstanding histories of neuroleptic use without incident [8]. Therefore, the duration of quetiapine therapy prior to the presentation should not be a deciding factor when considering NMS in a differential diagnosis. Quetiapine is commonly used off-label for sleep so even patients without known psychiatric histories may have the potential to develop the condition [9]. Coadministration of neuroleptics and selective serotonin reuptake inhibitors, such as in this patient suffering from depression with psychotic features, may increase the risk of developing NMS [10]. This calls particular attention to patients suffering from mixed depressive and psychotic disorders who may receive these medications concomitantly. At the same time, these patients are inherently predisposed to impulsivity and risk-taking behavior, such as suicide, by the virtue of their disease state [11-13]. Thus, patients with mixed depressive and psychotic disorders in the setting of overdose should prompt thorough medication reconciliation for NMS-inducing agents.

The exact mechanism of NMS is not fully understood, but contemporary theories revolve around dopamine’s inhibitory effect on centrally and peripherally acting neuronal networks that may help explain presentations of NMS in the absence of fever and rigidity. The prevailing theory of NMS pathophysiology suggests a disruption of dopamine receptor antagonism, specifically within the hypothalamus, leading to hyperthermia and dysautonomia [14]. Similar involvement in the nigrostriatal pathways may explain rigidity and tremor associated with NMS considering this pathway is highly implicated with similar Parkinsonian manifestations [2,15]. Direct and indirect toxicity of offending agents to peripheral tissues, such as skeletal muscles, may influence mitochondrial function leading to hyperthermia secondary to muscle rigidity [16]. Lastly, dopamine’s regulatory influence on autonomic control may lead to heightened sympathetic activity impacting temperature control and muscle tone [16]. In each scenario, rigidity and hyperthermia seem to be directly correlated. This patient experienced moderate rigidity in the absence of fever, an atypical presentation, with a mild disease progression. Taken together, this information suggests that patients with NMS who are afebrile may also have the potential to lack significant muscle rigidity, further reducing a major symptom indicator for the presence of NMS.

The timely diagnosis of NMS should be followed by the prompt discontinuation of inciting medication to prevent further symptom progression. The stage of NMS (mild, moderate, and severe) are used to inform the extent of supportive care and pharmacotherapy [17]. For instance, in this case, the patient’s symptoms were relatively mild, with acute renal failure that required close monitoring and muscle relaxers as needed, ultimately resolving over time. Progression to moderate or severe NMS in the presence of hyperpyrexia may warrant more intentional methods of cooling, with blankets or cooled saline. Severe cases may require direct muscle relaxers, such as dantrolene, or dopamine agonists such as bromocriptine. Monotherapy with dantrolene may actually increase mortality and should be avoided in patients with abnormal liver function due to increased risk of acute liver injury [18]. Once symptoms have resolved, approximately 30% of patients will experience a reoccurrence of symptoms [19]. Thus, it is important to slowly reintroduce an alternative antipsychotic medication in patients requiring therapy while remaining alert for signs and symptoms associated with this rare but serious condition.

## Conclusions

Neuroleptic malignant syndrome (NMS) is a life-threatening condition requiring prompt identification and diagnosis with an array of potential presenting symptoms. Certain symptoms, such as fever and muscle rigidity, are highly characteristic of the condition, however, atypical presentations have occurred. This case serves to illustrate an attenuated and atypical presentation of NMS in the setting of overdose with the second-generation antipsychotic quetiapine. Future studies should examine whether a significant relationship exists between fever status and outcome in patients with NMS.
